# Rac1 Deletion Causes Thymic Atrophy

**DOI:** 10.1371/journal.pone.0019292

**Published:** 2011-04-29

**Authors:** Lukas Hunziker, Salvador Aznar Benitah, Kristin M. Braun, Kim Jensen, Katrina McNulty, Colin Butler, Elspeth Potton, Emma Nye, Richard Boyd, Geoff Laurent, Michael Glogauer, Nick A. Wright, Fiona M. Watt, Sam M. Janes

**Affiliations:** 1 Centre for Respiratory Research, University College London, London, United Kingdom; 2 Internal Medicine, University Hospital Basel, Basel, Switzerland; 3 ICREA Researcher, Centre for Genomic Regulation (CRG) and UPF (Universitat Pompeu Fabra), Barcelona, Spain; 4 Centre for Cutaneous Research, Barts and The London, Queen Mary's School of Medicine and Dentistry, London, United Kingdom; 5 Wellcome Trust Centre for Stem Cell Research, Cambridge University, Cambridge, United Kingdom; 6 Department of Experimental Pathology, London Research Institute, Cancer Research UK, London, United Kingdom; 7 Department of Pathology and Immunology, Monash University Medical School, Prahran, Melbourne, Australia; 8 Faculty of Dentistry, University of Toronto, Toronto, Canada; 9 Histopathology Unit, London Research Institute, Cancer Research (UK), London, United Kingdom; 10 CR UK Cambridge Research Institute, Li Ka Shing Centre, Cambridge, United Kingdom; The National Institute of Diabetes and Digestive and Kidney Diseases, United States of America

## Abstract

The thymic stroma supports T lymphocyte development and consists of an epithelium maintained by thymic epithelial progenitors. The molecular pathways that govern epithelial homeostasis are poorly understood. Here we demonstrate that deletion of Rac1 in Keratin 5/Keratin 14 expressing embryonic and adult thymic epithelial cells leads to loss of the thymic epithelial compartment. Rac1 deletion led to an increase in c-Myc expression and a generalized increase in apoptosis associated with a decrease in thymic epithelial proliferation. Our results suggest Rac1 maintains the epithelial population, and equilibrium between Rac1 and c-Myc may control proliferation, apoptosis and maturation of the thymic epithelial compartment. Understanding thymic epithelial maintenance is a step toward the dual goals of *in vitro* thymic epithelial cell culture and T cell differentiation, and the clinical repair of thymic damage from graft-versus-host-disease, chemotherapy or irradiation.

## Introduction

The thymus is an epithelial organ responsible for T cell survival, maturation and selection [1]. It is formed by a cortex and medulla containing epithelial cells that are morphologically and functionally distinct [1], [2], [3]. Cortical epithelial cells support positive selection from immature CD4^+^/CD8^+^ thymocytes [4], [5], [6] while medullary epithelial cells enable induction of tolerance [7], [8]. A putative embryonic epithelial progenitor exists that is defined by cell surface expression of the glycoprotein MTS24 and EpCAM1 [9], [10], [11]. Transplantation experiments show that low numbers of MTS24^+^ epithelial cells taken from embryonic thymus, between gestational days 11.5–15.5, are capable of forming a fully functioning thymus with all epithelial subtypes, attract lymphoid progenitors and support CD4^+^/CD8^+^ lymphopoiesis [9], [10]. The use of MTS24 as a stem cell marker is however debated [12] but further progress has been made by lineage tracing single transplanted cells. Two studies using elegant lineage tracing techniques have established two populations capable of self-renewal and differentiation into medullary and cortical thymic epithelial cells (TECs) [13], [14]. One population is derived from embryonic day 12 (E12) thymic epithelium expressing EpCAM1 (these cells also express MTS24 and cytokeratin 5 (K5)) [14]. A second population capable of multipotent differentiation into both medullary and cortical epithelium is derived from post-natal medullary cells expressing cytokeratin 14 (K14), the K5 heterodimer [13]. This was demonstrated using lineage tracing driven by the Keratin 14 promotor. The thymic epithelial Keratin 14 expressing cells were typically thought confined to the thymic medulla however lineage tracing demonstrated colonies that were either medullary, cortical or mixed [13].

Several transcription factors required for thymic organogenesis have been identified [15], [16], [17], [18]. The best understood factor controlling murine thymic epithelial differentiation is *Foxn1*. *Foxn1* is thought to be required at the onset of differentiation and *Foxn1^−/−^* mice develop epithelial cysts without thymopoiesis [13], [18], [19]. *Foxn1^−/−^* epithelium appears immature and it has been suggested that it is required for the onset of normal thymic epithelial cell differentiation [18]. Replacement of *Foxn1* in single cells results in repopulation of small areas of thymic tissue capable of thymopoiesis [13].

In contrast, very little is known regarding the factors that maintain the thymic stem/progenitor cell compartment or mediate differentiation into the major thymic epithelial subsets of cortex and medulla. Recently it has been shown that p63 is dispensable for lineage commitment and differentiation during thymic organogenesis, but is required to maintain the proliferative potential of thymic epithelial progenitors [20], [21]. Furthermore, p63 appears to mediate survival of thymic epithelial stem cells in vivo by providing protection from programmed cell death [21]. It is predicted that the loss of stem cells would lead to the natural history of thymic involution, but it remains to be determined how the balance between proliferation and apoptosis is regulated during the process of ageing.

Rac1 plays essential roles in T-cell development and homeostasis [22]. For instance, pre-T cell differentiation and proliferation upon T cell antigen receptor (TCR) beta selection is dependent on Rac1 and its upstream activator Vav1 [23]. Interestingly, activation of Rac1 efficiently diverts pre-T cells from positive selection in the medulla into negative selection and subsequent deletion [24]. It has been postulated that Rac1 signals downstream of α6β4 integrin and p38MAPK in thymic epithelial cells to promote secretion of IL6 upon thymocyte contact [25]. However, the specific role of Rac1 in the epithelial compartment of the thymus has not yet been defined.

We wished to determine whether Rac1 has a role in the maintenance of the thymic epithelial cell compartment. We first deleted Rac1 in post-natal K14 expressing epithelial cells. Upon deletion these mice underwent a degree of thymic atrophy. We then found in an engraftment model that the deletion of Rac1 in K14 positive embryonic cells resulted in a failure of thymic organogenesis. K5 and K14 are heterodimers and hence we then used a constitutive model of K5 driven Rac1 deletion to confirm our results. The embryonic thymus at E12 is made up of a homogenous population of immature cells characterized by their expression of a series of proteins including EPCAM1, MTS24 and K5 and K8 [14], [26], [27]. Here we show embryonic deletion of Rac1 in K5 cells (which includes the progenitor populations [14]) leads in most cases to athymia or catastrophic thymic atrophy with loss of the medullary-cortical architecture. This atrophy may be due to a Rac1 mediated up-regulation of c-Myc leading to a global increase in apoptosis.

## Materials and Methods

### Ethics Statement and Experimental mice

All animal experiments were performed in compliance with Home Office and institutional guidelines. To lineage trace the K14 promoter K14CreER (kind gift from B. Stripp [28]) were crossed with CAG-CAT-eGFP (kind gift from J. Miyazaki [29]). Homozygous floxed Rac1 mice (Rac1^flox/flox^) and heterozygous for K14CreER (K14CreERxRac1^flox/flox^) or K5Cre (K5CrexRac1^flox/flox^) were generated as described previously [30], [31]. Briefly embryonic deletion of Rac1 was achieved by crossing Rac1^flox/flox^ mice [32] together with Keratin5-Cre (K5Cre) mice [33], [34]. Specifically, we first crossed K5Cre^het^ to Rac1^flox/flox^ to obtain K5Cre^het^xRac1^flox/wt^. We then crossed K5Cre^het^xRac1^flox/wt^ mice with Rac1^flox/flox^ to obtain K5Cre^het^xRac1^flox/flox^ knock-out mice (K5CrexRac1^flox/flox^). Deletion of Rac1 in adult thymus was obtained by crossing K14CreER^het^xRac1^flox/wt^ mice with Rac1^flox/flox^ mice (K14CreERxRac1^flox/flox^) [28], [30]. Cre-mediated deletion of Rac1 in K14CreERxRac1^flox/flox^ mice, and the stop signal in the K14CreER×CAG-CAT-eGFP reporter mice, was induced by weekly administration of 5 mg of intraperitoneal tamoxifen in 100 µl of peanut oil (Sigma) for three weeks. Thymic grafts were placed in female ICRF nu/nu mice kept in filtered sterile cages.

### Foetal Thymic Organ Cultures

Thymic lobes were removed from E15.5 embryos and cultured in complete medium (RPMI with 10% FCS, 2 mM glutamine, 10 mM HEPES), with or without 100 nM 4-hydroxy-tamoxifen. Lobes were then removed from culture and prepared for frozen sectioning.

### Antibodies

Antibodies against Rac1 (Clone 23A8, Upstate Biotechnology, 1∶100 dilution), Ki67 (Novacastra, 1∶400 dilution), Keratin 14 (Babco, 1∶1000), Keratin 5 (Abcam, 1∶1000), Keratin 8 (Abcam, 1∶1000), phospho-serine 20-PAK2 (US Biologicals, 1∶100), GFP (ab5450, Abcam, 1∶500), c-Myc (N262, Santa Cruz, 1∶100), anti-ER (HL7, 1∶100), and MTS24 (1∶100) [9] were used for immunofluorescence as previously described [28], [30]. Secondary antibodies for immunofluorescence conjugated to Alexa-488, Alexa-594 and Alexa-633 were purchased from Molecular Probes and used at a 1∶400 dilution. Nuclei were counter- stained with DAPI or To-Pro-3 (Invitrogen). APC conjugated anti-mouse CD8, FITC conjugated anti-mouse CD3 and PE conjugated anti-mouse CD4 were used for splenic and thymic cell flow cytometry (BD Pharminogen).

### Primary Thymic Epithelial Cell Harvest and Kidney Capsule Grafting

Primary thymic epithelial cells were harvested as described elsewhere [35]. Cells were derived from E13.5 stage mouse embryos. 2000 MTS24^+^ and 25000 MTS24^−^ cells were sorted by fluorescence activated cell sorting. Cells were resuspended in a small volume of RPMI and placed on a fragment of filter paper for 24 hours with or without 100 nM 4-hydroxy-tamoxifen (Sigma). The paper and cells were then implanted beneath the kidney capsule of ICRF nu/nu mice and harvested after 8 weeks. The thymus was taken for immunohistochemistry and the spleens for flow cytometry.

### Immunofluorescence Staining

Thymus was embedded in OCT compound (Bayer) and 5 mm frozen sections cut. Frozen sections for Rac1 staining were thawed at room temperature, fixed in 4% paraformaldehyde/2% acetic acid in PBS for 30 minutes followed by 2 minutes in ice cold ethanol/acetic acid (95/5). All other sections were fixed in 4% paraformaldehyde for 20 minutes at room temperature and permeabilised for 5 minutes in 0.3% Triton X 100. Staining was analysed using a Leica TCSNT confocal microscope. Proliferating epithelial cells were only counted if the epithelial marker clearly surrounded a Ki67 positive nucleus.

### MTS24 FACS sorting

Thymic epithelial cell isolation was performed as described [9]. Briefly, five to eight small incisions were made in each isolated thymic lobe. The tissue was then stirred for 1 h at 4°C in serum-free RPMI-1640. Remaining tissue aggregates were digested to single-cell suspension by incubation at 37°C with 0.01% DNase I and 0.15% collagenase D. Cells from the suspension were washed in cold FACS buffer and subsequently stained with anti-MTS24 antibody and sorted using a FACScalibur II sorter.

### Statistics

Statistical analysis was performed with the one-tailed Mann-Whitney U test using GraphPad Prism3.00 for Windows (GraphPad Software). P<0.05 was interpreted as statistically significant.

## Results

### Keratin and Rac1 expression in murine thymus

**Figure 1:**
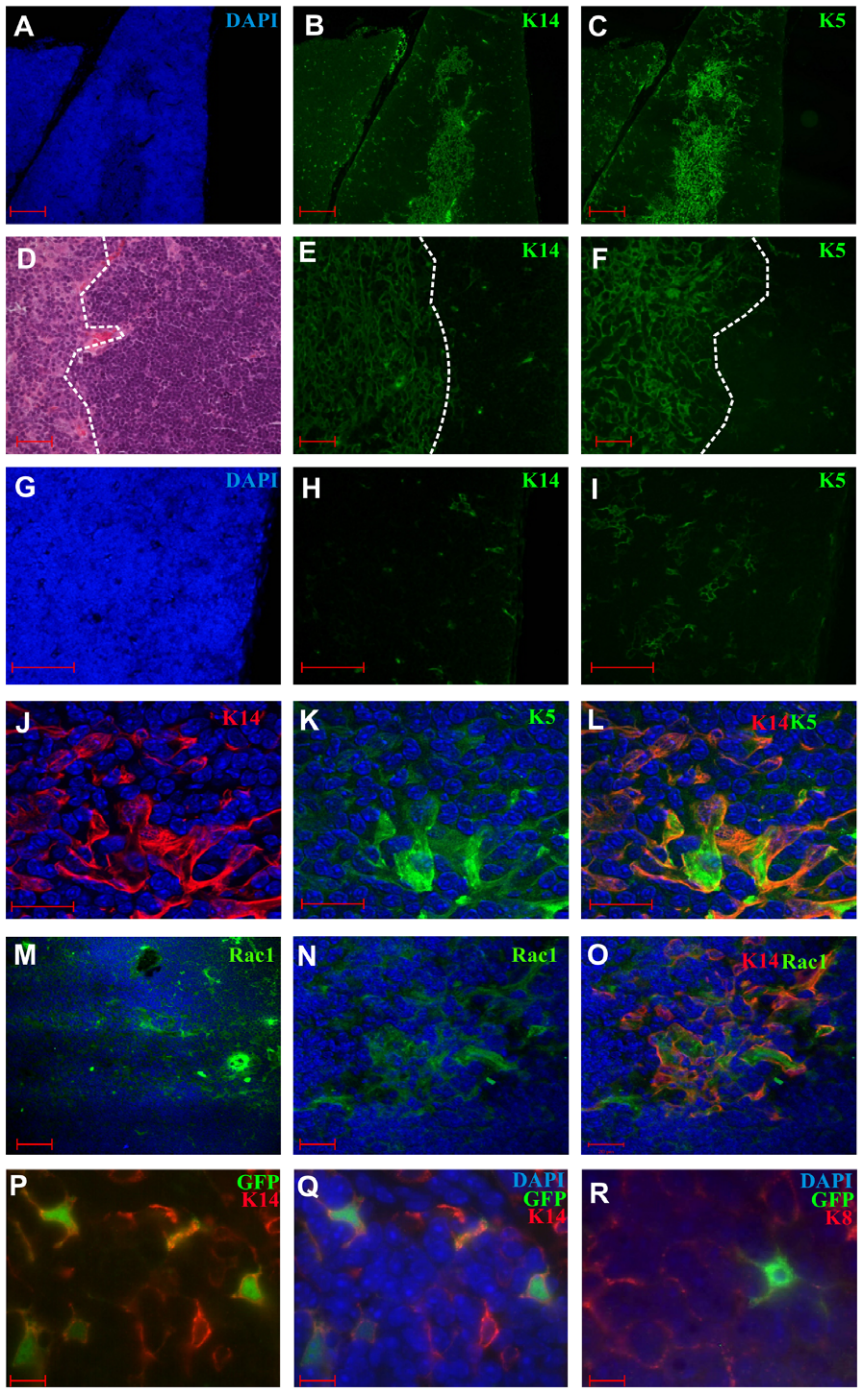
Keratin and Rac1 expression in the thymus. (A–F and J–L) K14 and K5 largely co-localise in medullary epithelium; dotted lines in D–F denote medullary-cortical boundary. (G–I) Thymic cortex with scattered K14 and K5 positive cells. (M–O) Rac1 staining is widespread in the thymus (epithelial cells and thymocytes (M)) but the brightest Rac1 staining co-localises with K14 positive epithelial cells of the medulla, (N and O). (P and Q) demonstrate K14 positive epithelial cells that are GFP positive in K14 lineage tracing analysis, but no GFP positive cells in the K8 stained cells (R). Scale bars (A–C) 100 µm, (D–I, M and R) 50 µm, (J–L, N and O) 20 µm, (P–R) 5 µm.

Medullary TECs express K14 and K5 throughout the thymic medulla (Figure 1A–F and J–L) [10]. K14 and K5 expressing cells are not exclusive to the medulla however with positive cells scattered throughout the cortex (Figure 1G–I). Rac1 staining shows expression in the majority of cells within the thymus (Figure 1M) but the immunofluorescence is brightest in epithelial cells (Figure 1N and O). Therefore, Rac1 is widely expressed throughout the thymus although brightest in the medullary epithelia.

In the following experiments we wished to examine the effects of deleting Rac1 in K14/K5 positive thymic epithelial cells. To this end we used a Cre/loxP system targeted by the human K14 and K5 promoters. To demonstrate the targeting using this system we crossed K14CreER mice with the CAG-CAT-GFP reporter mice. Hence on activation of the ER receptor the Cre-recombinase will activate the expression of GFP. Mice received weekly injections of 5 mg tamoxifen for three weeks. Immunofluorescent staining of thymi from four mice confirmed expression of GFP in K14 positive cells but not in K8 cells (Figure 1P–R). GFP was detected in 22.2%±4.5 (SD) of K14 positive cells with double immunofluorescence (n = 4 mice).

### Adult thymic Rac1 depletion results in loss of thymic tissue

We wished to investigate whether Rac1 had a role in adult thymic epithelial cell homeostasis. For these experiments we used K14CreERxRac1^flox/flox^ mice. We compared mice treated with tamoxifen (activating Cre recombinase) to control mice consisting of untreated K14CreERxRac1^flox/flox^ mice or litter mates that were Cre negative with and without tamoxifen.

**Figure 2:**
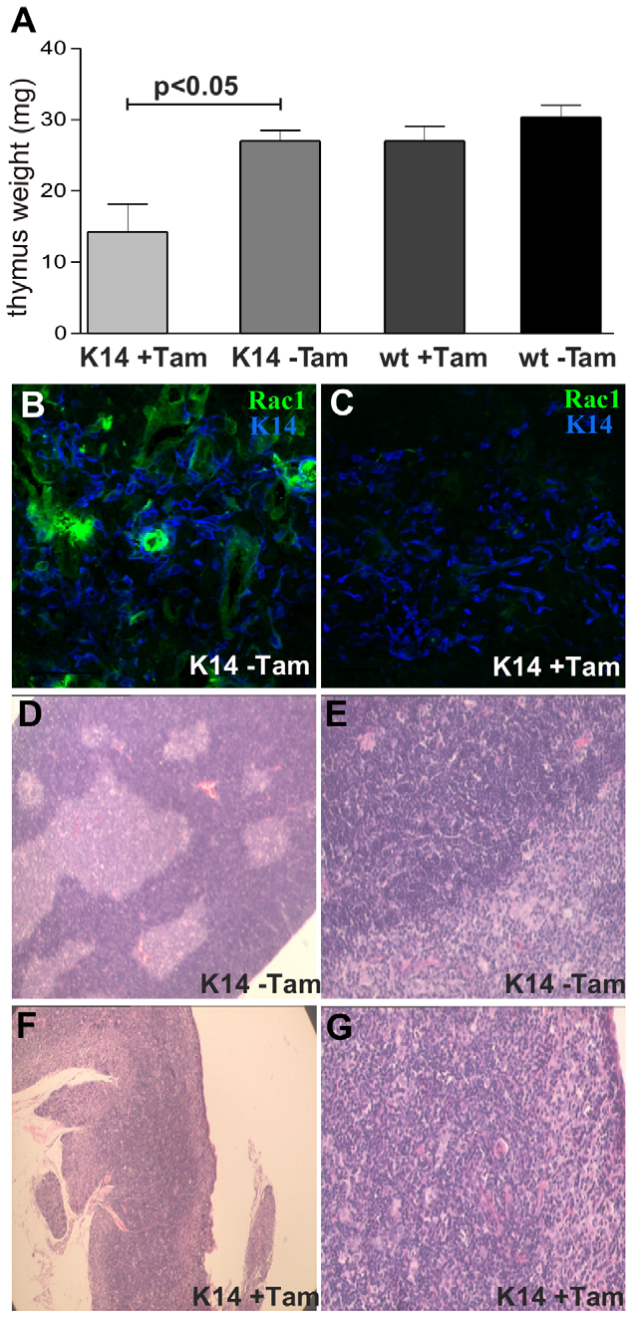
Effects of adult epithelial Rac1 deletion on thymus homeostasis and architecture. (A) Reduced weight of tamoxifen-treated K14CreERxRac1^flox/flox^ (K14+Tam) compared to controls: no tamoxifen treated K14CreERxRac1^flox/flox^ (K14−Tam), tamoxifen treated and untreated no Cre litter mates (wt+Tam and wt−Tam respectively); p<0.05). (B and C) Rac1 immunofluorescence demonstrating Rac1 deletion in the K14+Tam (C) compared to untreated control (B). (D and E) Normal medullary-cortical architecture of untreated K14CreERxRac1^flox/flox^ mice (K14−Tam). (F and G) Loss of distinct medullary/cortical boundaries after three weeks in tamoxifen treated K14CreERxRac1^flox/flox^ mice (K14+Tam). (B and D ×4 and C and E ×10).

After three weeks of tamoxifen treatment we observed a significant decrease in thymic weight in 8 week old K14CreERxRac1^flox/flox^ mice (mean ± SD; 14.3 mg±7.8) compared to untreated K14CreERxRac1^flox/flox^ mice (27 mg±2.7; (p<0.05)), and tamoxifen treated and untreated Cre negative littermates (27.0 mg±3.6 (p<0.05), and 30.3 mg±2.9 (p<0.05) respectively) (n = 5 per group) (Figure 2A). Rac1 deletion was confirmed by immunofluorescence (Figure 2B and C). Histological analysis of tamoxifen treated K14CreERxRac1^flox/flox^ mice revealed destruction of the medullary-cortical architecture with loss of medulla compared to littermate controls (Figure 2D–G and Fig. S1 A–H). FACS analysis of peripheral T cells from spleens showed normal distribution of CD4^+^ and CD8^+^ T cells in tamoxifen treated K14CreERxRac1^flox/flox^ mice (data not shown), however there was a block in thymic T cell maturation with an increase in the number of CD4^−^/CD8^−^ premature T cells (Figure S1 M–O and Table S1).

### Rac1 is required for thymic organogenesis

**Figure 3:**
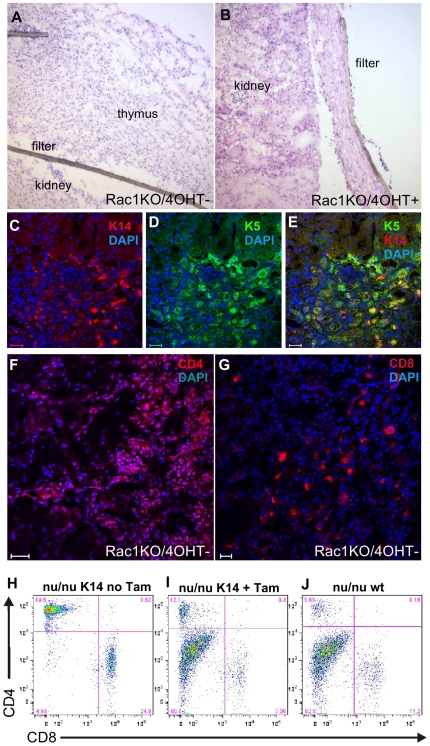
Rac1 deletion results in the inhibition of thymic ontogeny. 4-hydroxy-tamoxifen treatment of K14CreERxRac1^flox/flox^ MTS24^+^ E13.5 thymic epithelial cells (Rac1KO/4OHT+) blocks the generation of a thymic microenvironment after heterotopic transplantation under the kidney capsule (B) compared to untreated controls (Rac1KO/4OHT− shown) (A). (C–E) K5 and K14 staining confirming the presence of thymic medullary epithelial cells in control cells (shown are untreated MTS24^+^ K14CreERxRac1^flox/flox^ (Rac1KO/4OHT−))^which^ were capable of generating a functional thymic microenvironment as indicated by the presence of CD4^+^ (C) and CD8^+^ cells. (A and B ×20); Scale bars (C and D) 20 µm. FACS analysis of splenocytes derived from nude mice grafted with K14CreERxRac1^flox/flox^ MTS24^+^cells in absence of Tamoxifen showed peripheral CD4 and CD8 positive populations confirming the function of the thymic grafts (H) while with tamoxifen treated mice (I) showed no maturation of peripheral lymphocytes consistent with untansplanted nude mice (J). The dot plots show cells labelled with anti-CD4 and anti-CD8 antibodies gated on a CD3^+^ population.

The thymic atrophy induced by Rac1 deletion in adult K14 positive cells led us to predict that we could block thymic organogenesis by embryonic deletion of Rac1. To do this we initially used MTS24 expression to fluorescence activated cell sort for embryonic (E13.5) thymic epithelial cells from wild-type and K14CreERxRac1^flox/flox^ mice. A majority of epithelial cells are MTS24 positive in the E13.5 thymus [36]. Purified MTS24^+^ and MTS24^−^ cells were incubated for 24 hours with or without 4-hydroxy-tamoxifen (4OHT) (or vehicle) and underwent heterotopic transplantation under the kidney capsule of athymic ICRF nude mice. 4OHT treated wild-type MTS24^+^ (4 of 6 mice, not shown) and non-treated K14CreERxRac1^flox/flox^ MTS24^+^ cells (5 of 6, Figure 3A) regenerated a functional thymic microenvironment as shown by K5 and K14 expression (Figure 3C–E), the generation of CD4^+^ (Figure 3F) and CD8^+^ cells (Figure 3G) and flow cytometry of the spleen showing mature T cells (CD3^+^CD4^+^ and CD3^+^CD8^+^ T cells) derived from the engrafted thymus in the these nude mice (Figure 3H). However, 4OHT treated, and therefore Rac1 depleted K14CreERxRac1^flox/flox^ cells were incapable of thymic organogenisis (0 of 6, Figure 3B) and had no evidence of mature T cells (Figure 3I) comparable to NOD/SCID untransplanted controls (Figure 3J) and Table 1. MTS24^−^ cells were also not able to regenerate a thymic microenvironment (0 of 6, not shown). This experiment confirms that deletion of Rac1 from embyronic thymic epithelia leads to failure of thymic organogenesis.

**Table 1:** Proportions of CD3 and CD4 positive cells in the spleens of transplanted NOD/SCID mice.

CD4/CD8 status	ICRF nu/nu+K14 no tamoxifen	ICRF nu/nu+K14+tamoxifen	ICRF nu/nu untreated
CD3+CD4+CD8+	0.72%+/−0.14	0.12%+/−0.1	0.3%+/−0.2
CD3+CD4+CD8−	67.9%+/−2.2	5.8%+/−3.8	5.1%+/−2.7
CD3+CD4−CD8+	24.9%+/−0.1	5.7%+/−2.7	12.8%+/−5.0
CD3+CD4−CD8−	6.5%+/−2.1	88.5%+/−5.6	81.9%+/−7.3

### Embryonic Rac1 depletion results in catastrophic loss of thymic tissue

**Figure 4:**
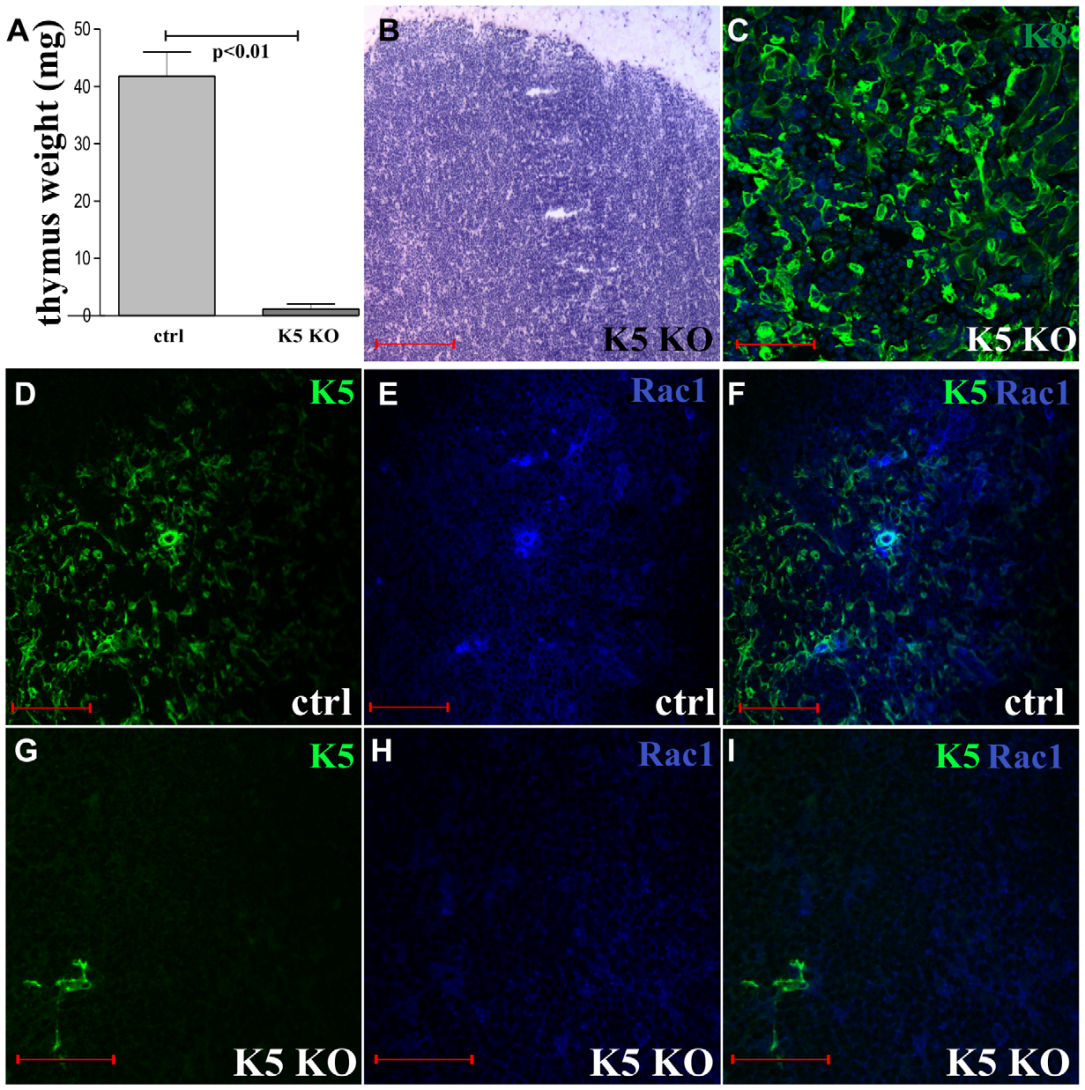
Effects of embryonic Rac1 deletion on thymus ontogeny. (A) Reduced weight of K5CrexRac1^flox/flox^ (K5 KO) thymus compared to Cre negative litter mate controls (ctrl) (error bars are Standard Errors). (B and C) Destruction of distinct medullary/cortical boundaries in K5CrexRac1^flox/flox^ mice (K5-KO) (B) leaving predominantly K8 positive cortex (C). (D–I) Cre negative littermates (ctrl, D–F) have normal Rac1 and K5 expression compared to reduced expression in K5CrexRac1^flox/flox^ (K5-KO) mice (G–I). (B) ×10 and (C) ×20. Scale bars 50 µm.

In order to confirm the importance of Rac1 in the epithelial homeostasis of the thymus, we next deleted Rac1 in embryonic thymic epithelial cells using a constitutive model. In these experiments K5CrexRac1^flox/flox^ mice (K5 is the K14 heterodimer) were compared to their Cre negative littermates. Of 21 K5CrexRac1^flox/flox^ mice born, 16 were completely athymic and 5 had thymic remnants. The weight of K5CrexRac1^flox/flox^ thymus was 1.04 mg ±3.28 (mean ± SD) (all mice, athymic included) compared to 41.8 mg±7.3 of the litter mate controls (p<0.01) (Figure 4A). Histological analysis of the five K5CrexRac1^flox/flox^ mice with thymic tissue at 6 weeks revealed complete loss of the medulla compared to littermate controls (Figure 4B and 4C). Immunofluorescence demonstrated a loss in K5 expressing thymic epithelial cells with a correspondingly depleted Rac1 compared to litter mate controls (Figure 4D–I and Figure S1 I–L). Immature thymocytes similar to the K14CreERxRac1^flox/flox^ tamoxifen treated mice were again demonstrated (Supplementary Figure S1M–O).

These results show that Rac1 depletion in both mouse models leads to the loss of thymic epithelial cells and destruction of the normal thymic architecture.

### Rac1 depletion leads to a loss of epithelial proliferation, increased apoptosis and high c-Myc immunofluorescence

**Figure 5:**
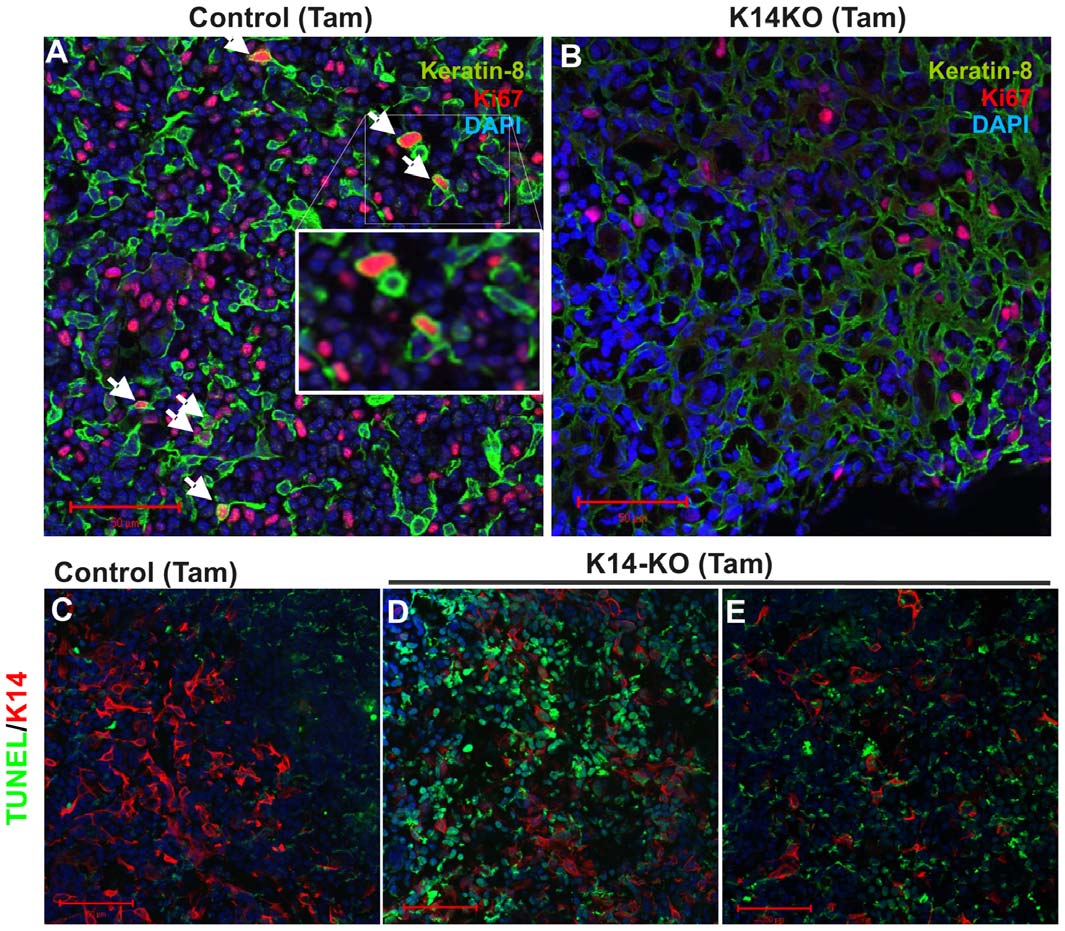
Rac1 deletion results in decreased proliferation and increased apoptosis. (A and B) Tamoxifen treatment results in reduced Ki67 positive epithelial and non-epithelial cells in K14CreERxRac1^flox/flox^ mice (K14−KO) compared to litter mate controls (Control Tam). Representative immunofluorescence pictures with arrows indicating co-localization between Keratin-8 (green) and Ki67 (red) in litter mate controls only. (C–E) Increased apoptosis in both K14^+^ (red) and K14^−^ cells in tamoxifen-treated K14CreERxRac1^flox/flox^ mice (medulla: day 12 (D), day 20 (E) compared to tamoxifen treated Cre negative litter mates (C). Scale bars 50 µm.

Rac1 deletion led to a clear reduction in the proportion of Ki67 positive cells across the thymus, including epithelial cells, in tamoxifen-treated K14CreERxRac1^flox/flox^ mice compared to controls (total proliferating cells (thymocytes and epithelial cells) 7.0%±5.0 compared to 18.5%±2.0 (p<0.05); and proliferating epithelial cells 0% compared to 11%±3.6 (p<0.05) (Figure 5A and 5B). Loss of proliferation was accompanied by an induction of apoptosis, measured by TUNEL assay, in both epithelial and non-epithelial cells (probably precursor T cells). In littermate controls 6.5%±2.4 of total cells were TUNEL positive (Figure 5C) compared to K14CreERxRac1^flox/flox^ mice tamoxifen-treated for 12 days (63.8%±8.3; p<0.05, Figure 5D) and 20 days (70.5%±7.8; p<0.05, Figure 5E).

Deletion of Rac1 in keratin-14 or keratin-5 positive epidermal basal cells results in progressive loss of the entire epidermal compartment [30], [31]. Rac1 null keratinocyte stem cells irreversibly commit to terminal differentiation (a process with strong similarities to apoptosis [37]) in a PAK2- and c-Myc-dependent manner [30]. We hypothesised this was also the case in the thymus. Very few c-Myc positive cells could be detected in the thymus by immunofluorescent staining of wild type 6 week old litter mate mice however the remnants of thymic epithelium of K5CrexRac1^flox/flox^ mice showed a marked increase (Supplementary Figure S2A–D). Similarly, un-treated fetal thymic organ cultures (FTOC) of K14CreERxRac1^flox/flox^ mice showed low expression of c-Myc (17.9%±7.3; Supplementary Figure S2E), however K14CreERxRac1^flox/flox^ derived FTOC in the presence of 4OHT resulted in up-regulation of c-Myc immunofluorescent nuclei (51.1%±8.4; p<0.05; Figure S2F). These data suggest the possibility that the thymic epithelium may have similar homeostatic mechanisms to the epidermis and is an avenue we are further investigating.

## Discussion

Using two different transgenic mice we provide evidence that Rac1 is important in thymic epithelial cell homeostasis. To determine whether Rac1 is required for adult thymus homeostasis we used a model of conditional deletion of Rac1 in post-natal mice. Here we targeted K14 expressing cells. K14 is expressed in medullary epithelial cells which include a population shown to include adult thymic progenitor cells [13]. After Rac1 deletion we saw a reduction in thymic size and destruction of the medullary-cortical architecture. In the K5CrexRac1^flox/flox^ transgenic mouse (K5 is the K14 heterodimer), where Rac1 is deleted after embryonic expression of K5 (E12.5), 16 of 21 mice were athymic and the remaining 5 showed a greatly reduced thymic size. In the 5 mice that had a remnant thymus there was gross destruction of the normal medullary-cortical architecture with loss of the medulla.

EpCAM1 and MTS24 are expressed by embryonic day 12 (E12) and the K5/K14 heterodimer around E12.5 [9]. In the embryo, MTS24^+^K5^+^ cells are believed to be progenitors of both cortical and medullary thymic epithelial cells. In our experiments not all K5CrexRac1^flox/flox^ were however athymic. Due to the slightly delayed expression of K5, and the presence of a population that is MTS24^+^/K8^+^/K5^−^, it is possible that a delayed or inefficient deletion of Rac1 in thymic stem cells led to the generation of a small thymus, or the expansion of the MTS24^+^/K8^+^/K5^−^ population. In the majority of mice however, it appears that the deletion of Rac1 induces global epithelial cell differentiation or apoptosis.

We used two models of Rac1 deletion to underline the importance of Rac1 in thymic epithelial cell homeostasis. The K14 promotor driven system is conditionally active allowing us to target K14 positive cells in the adult thymus with the majority of K14 positive cells in the medulla. Recently it has been demonstrated that a population of postnatal K14 expressing cells can act as adult progenitors and form medullary, cortical or mixed cell daughters [13]. While our experiment may target this small population of adult progenitors of the medulla and cortex, the activation of Cre after tamoxifen administration in our system of Rac1 deletion activated Cre in a large number of K14 expressing cells resulting in the rapid phenotype demonstrated on Cre activation.

The K5 transgenic system is constitutive and will be activated in the embryonic thymus. It has been demonstrated that K5 co-localises with K8 and the putative thymic progenitor marker MTS24 at E12.5. In the adult, K5 positive cells are largely located within the medulla. However a significant population of adult K5 positive cells lie within the cortex [26]. These adult cortical thymic K8+K5+ cells contain precursors that give rise to the major cortical K8+K5− subset. We therefore anticipate that the constitutive deletion of Rac1 in K5 expressing cells from E12.5 will target a wide selection of thymic epithelial cells including both embryonic thymic progenitors, differentiated medullary cells and adult cortical precursors.

Additional evidence lending support to Rac1 deletion causing failure of thymic ontogeny seen in the K5CrexRac1^flox/flox^ mice was gained by using heterotopic transplantation of embryonic thymic cells from the K14CreERxRac1^flox/flox^ mice. We used MTS24 expression to sort epithelial cells and deletion of Rac1 blocked the regeneration of a new thymic microenvironment when transplanted under the kidney capsule. Importantly the MTS24 negative cell population used in our controls includes non-epithelial cells and hence differs from that previously used by Rossi and colleagues where a second epithelial marker was used (Epcam1) to ensure MTS24- epithelial cells were used [12]. These experiments combined with the K14 litter mates controls supplied important controls for Cre expression and tamoxifen treatment.

A recent paper has questioned whether the MTS24 marker uniquely identifies a thymic progenitor subpopulation with the ability to repopulate functional thymic epithelium [12]. These differences from previous reports [9], [10] are likely due to different sorting strategies and differing numbers of cells implanted. Importantly, in our studies we implanted a low number of cells that were confirmed simply as epithelial based on MTS24 expression. We have demonstrated that Rac1 expression is required for this subset of epithelial cells to regenerate the thymic microenvironment.

It has been proposed that Rac and Myc represent a global stem cell regulatory axis [30]. In the epidermis Rac1 deletion leads to loss of stem cells while acute Myc over-expression promotes epidermal and hematopoietic differentiation, possibly through disruption of cellular adhesion [30], [38], [39], [40], [41]. Conversely, in the gut Myc determines self renewal while loss of Rac1 triggers differentiation [42]. In other models sustained induction of Myc leads to tumor development [43]. Hence it is known from other epithelial systems that Myc regulation is tightly controlled to avoid differentiation of stem cells or tumorigenesis. To determine whether a Rac1-Myc axis may be involved in thymic epithelial homeostasis, we showed in Fetal Thymic Organ Cultures derived from K14CreERxRac1^flox/flox^ mice and six week old K5CrexRac1^flox/flox^ thymus that Rac1 deletion leads to an increase in c-Myc expression. While this is not conclusive evidence that the same regulatory pathways are operational in the thymus as the skin we believe this will be an interesting line of future investigation.

In conclusion deletion of Rac1 results in the failure of thymic ontogeny in embryos and thymic atrophy in adults. Understanding mechanisms of thymic stem cell maintenance may help the development of therapies for patients with thymic developmental defects, or reverse damage from aging, chemo or radiotherapies. Further, maintenance of thymic epithelium ex vivo may allow in vitro generation of differentiated T cells.

## Supporting Information

Figure S1 Distribution of keratins in vivo after Rac1 deletion. (A–D) K8, K5 and K14 localisation in wild type thymus compared to K14CreERxRac1^flox/flox^ (E–H) and K5CrexRac1^flox/flox^ (I–L). Scale bars 50 µm. (M–O) Impaired thymic selection with increase of the CD4/CD8 double negative population in tamoxifen treated K14CreERxRac1^flox/flox^ mice (N) and K5CrexRac1^flox/flox^ (O) compared with tamoxifen treated wild type mice (M).
(TIF)

Figure S2 Loss of Rac1 results in up-regulation of c-Myc. (A–D) Tamoxifen treatment of K5CrexRac1^flox/flox^ results in increase immunofluorescence staining of c-myc in 6 week old remnant thymi (A–D). Addition of tamoxifen to K14CreERxRac1^flox/flox^ (K14+Tam) derived Fetal Thymic Organ Cultures causes increased c-Myc expression (E and F) compared to controls (K14 no Tam) (B and C). Scale bars 50 µm.
(TIF)

Table S1 Proportions of CD3 and CD8 positive peripheral T cells from spleens tamoxifen treated wild type, tamoxifen treated K14KO and K5KO mice.
(DOCX)
