# Correction: Rac1 Deletion Causes Thymic Atrophy

**DOI:** 10.1371/annotation/5c665e96-0303-438f-afb0-c257c3481692

**Published:** 2011-06-02

**Authors:** Lukas Hunziker, Salvador Aznar Benitah, Kristin M. Braun, Kim Jensen, Katrina McNulty, Colin Butler, Elspeth Potton, Emma Nye, Richard Boyd, Geoff Laurent, Michael Glogauer, Nick A. Wright, Fiona M. Watt, Sam M. Janes

The second author's name appears incorrectly in the citation. The correct citation is: Hunziker L, Benitah SA, Braun KM, Jensen K, McNulty K, et al. (2011) Rac1 Deletion Causes Thymic Atrophy. PLoS ONE 6(4): e19292. doi:10.1371/journal.pone.0019292 

