# Supplementary figures and images for: Rac1 Deletion Causes Thymic Atrophy
Source: PLoS One. 2011 Apr 29;6(4):e19292. doi: 10.1371/journal.pone.0019292 (PMC3084817; doi:10.1371/journal.pone.0019292)

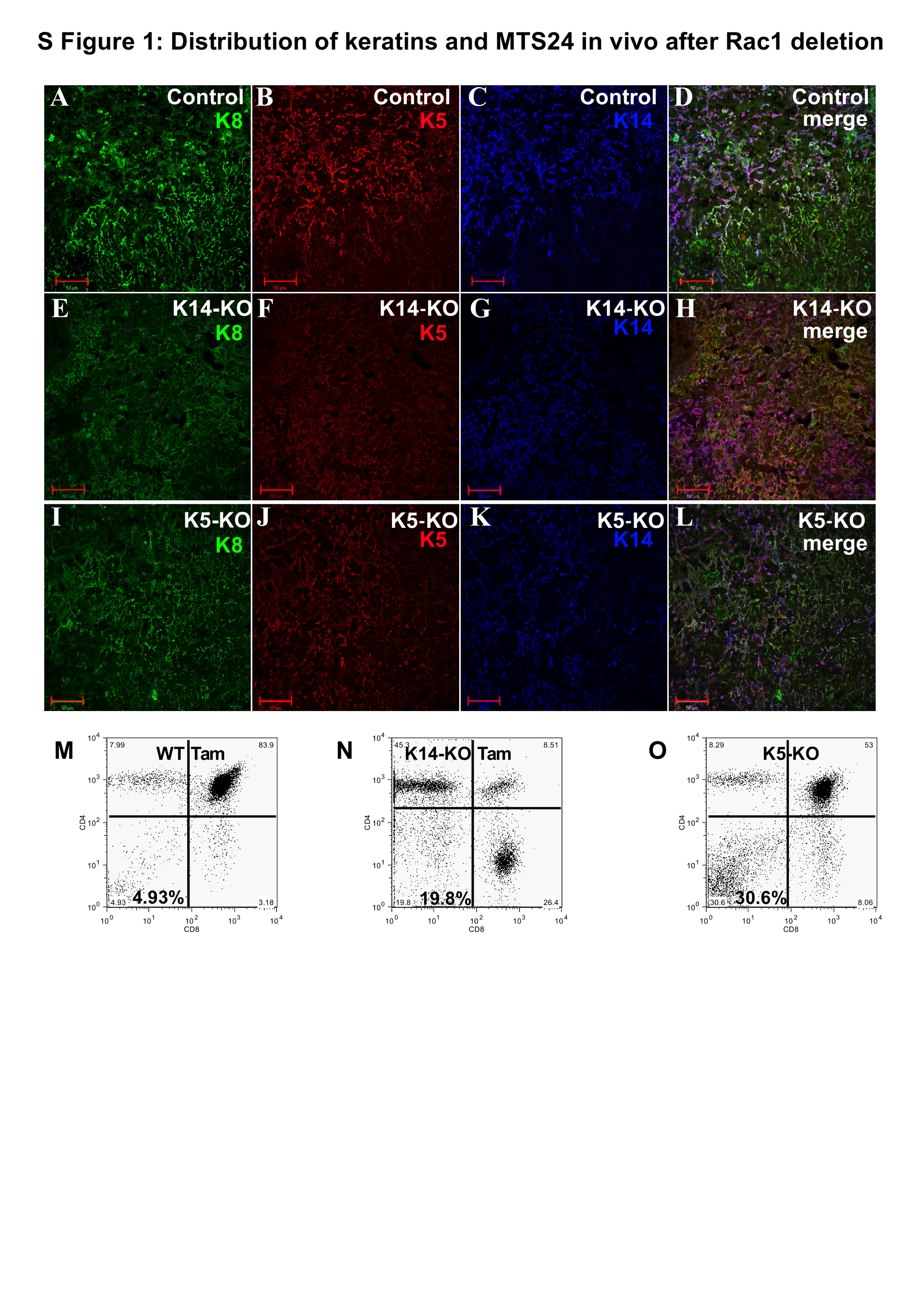

Supplement: Figure S1 — Distribution of keratins in vivo after Rac1 deletion. (A–D) K8, K5 and K14 localisation in wild type thymus compared to K14CreERxRac1flox/flox (E–H) and K5CrexRac1flox/flox (I–L). Scale bars 50 µm. (M–O) Impaired thymic selection with increase of the CD4/CD8 double negative population in tamoxifen treated K14CreERxRac1flox/flox mice (N) and K5CrexRac1flox/flox (O) compared with tamoxifen treated wild type mice (M). (TIF) [file pone.0019292.s001.tif]

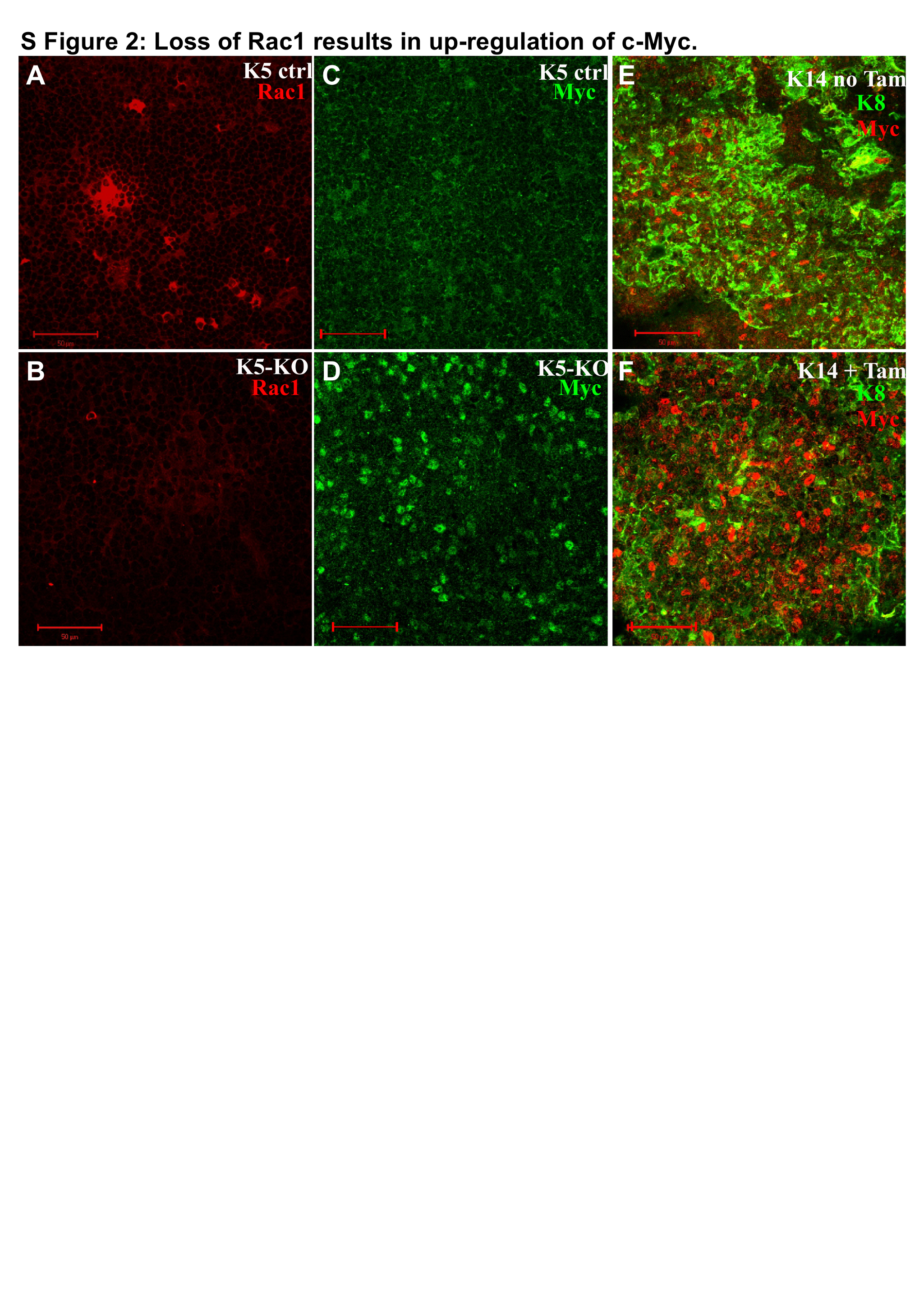

Supplement: Figure S2 — Loss of Rac1 results in up-regulation of c-Myc. (A–D) Tamoxifen treatment of K5CrexRac1flox/flox results in increase immunofluorescence staining of c-myc in 6 week old remnant thymi (A–D). Addition of tamoxifen to K14CreERxRac1flox/flox (K14+Tam) derived Fetal Thymic Organ Cultures causes increased c-Myc expression (E and F) compared to controls (K14 no Tam) (B and C). Scale bars 50 µm. (TIF) [file pone.0019292.s002.tif]
