# Supplementary material for: Rac1 Deletion Causes Thymic Atrophy
Source: PLoS One. 2011 Apr 29;6(4):e19292. doi: 10.1371/journal.pone.0019292 (PMC3084817; doi:10.1371/journal.pone.0019292)
Supplement: Table S1 — Proportions of CD3 and CD8 positive peripheral T cells from spleens tamoxifen treated wild type, tamoxifen treated K14KO and K5KO mice. (DOCX) [file pone.0019292.s003.docx]

| CD4/CD8 status | Wild type +tamoxifen | K14-KO +tamoxifen | K5-KO +tamoxifen |
| --- | --- | --- | --- |
| CD4+CD8+ | 84.6% +/- 2.5 | 7.1% +/- 4.4 | 51.4% +/- 14.4 |
| CD4+CD8- | 2.9% +/- 1.1 | 41.2% +/- 12.7 | 8.6% +/- 3.4 |
| CD4-CD8+ | 8.0% +/- 1.3 | 38.1% +/- 11 | 21.3% +/- 17.7 |
| CD4-CD8- | 4.5% +/- 1.7 | 13.7% +/- 6.6 | 18.7% +/- 11 |
